# Hospital and Health News

**Published:** 1923-09

**Authors:** 


### HOSPITAL AND HEALTH NEWS

### The Leicester Infirmary Extension.

The new wing and other buildings at the Leicester Royal Infirmary will probably be opened next month.

### No More Advertisement Revenue.

The new forms of National Health Insurance certificates will not bear trade advertisements on the back.

### Home Grown Potatoes.

Two plots, comprising in all 600 square yards, have been sown with potatoes for the benefit of the Newcastle hospitals by the Walker Allotments Association.

### Unfounded Charges.

The Special Committee of the Wolverhampton Town Council appointed to investigate has dismissed as unfounded the charges recently brought against the Borough Fever Hospital by Mrs. Sproson.

### Manchester's Seaside Hospital.

The Public Health Department at Manchester has prepared a scheme for a children's hospital at Abergele to contain 200 beds, for which the sanction of the Ministry of Health is being sought.

### Resignation of Mr. C. R. W. Offen.

Mr. C. R. W. Offen, who has been house governor at the Warneford Hospital, Leamington, since October, 1917, has handed in his resignation and will relinquish his post as from September 8.

### A Memorial Operating Theatre.

A new operating theatre is to be built at the Buxton and District Cottage Hospital in memory of the late Dr. Joshua J. Cox, who from 1914-17 was P.R.M.O. for the Manchester area, and Commissioner of Medical Services for the N.W. region 1917-18.

### A Hospital Meteorologist.

The Duke of Devonshire, patron of the Devonshire Hospital, Buxton, has presented on behalf of the hospital and the town council, a barograph and a cheque to Mr. W. Pilkington, who has been honorary meteorologist to the institution for twenty-six years.

### League of Mercy's Help for Hospitals.

For the year 1922 £15,000 was handed over by the League of Mercy to King Edward's Hospital Fund, making a total of £339,034 since the League was started in 1899. In addition to this, £5,390 was awarded in grants to extra-metropolitan hospitals in 1922.

### Streatham Home for Incurables.

The Prince of Wales will preside at a Festival Dinner in aid of the British Home and Hospital for Incurables, Streatham, at the Savoy Hotel on November 27. A lady has promised £1,000 to name a bed, expressing the hope that four others will do the same, so that a room may be named after the Prince of Wales.

### The late Miss Pawling.

The death of Miss Pawling, founder and for thirty-two years sister-in-charge of the Children's Home Hospital, Hadley, Barnet, is a severe loss to the institution. The present building, which accommodates twenty children recovering from operations, was opened about thirteen years ago. Miss Vale, an old member of the staff, has been appointed to succeed Miss Pawling.

### Healthiest London.

The London death-rate in the first week of August was the lowest ever recorded in the history of the capital. It was 7.9 per 1,000 of the population. Curiously enough the former lowest rate occurred about the same time last year—in the week ended August 19. It was 8.1 per 1,000. During the influenza epidemic of January, 1922, the death-rate was 31.8 per 1,000.

### A Lethal Weapon.

A London doctor recently visited Dublin. On landing he saw other passengers putting their hands up, so he followed suit. A Customs official searched him for hidden weapons. He found one, and called for assistance. Three officials examined it meticulously, and finally passed its owner. The weapon was a stethoscope.

### "Idozan."

Idozan, a colloidal iron concentrate, is being found very valuable by medical men in the treatment of cases of anæmia. It is pleasant to take and neither discolours the teeth nor upsets the system. It is usually prescribed alone, but may be combined with vegetable tinctures, though it is incompatible with alkaline salts. Idozan is manufactured by Messrs. Charles Zimmerman, of 9 and 10, St. Mary-at-Hill, E.C. 3, who have received many testimonials from doctors as to its efficacy.

### Mr. George Robey's Hospital Performance.

Mr. George Robey, dressed as Sancho, whose part he plays in a film version of *Don Quixote*, has assisted the great appeal being made on behalf of the Wolverhampton General Hospital. He toured the wards and then gave a short performance on the roof for the amusement of the great crowd in the street, to whom he warmly commended the advantages of the voluntary system.

### Co-operators help Keighley Hospital.

By the narrow majority of two the Keighley Co-operative Society has agreed to make a grant of £1,000 from its reserve fund to the Keighley Victoria Hospital Bazaar Fund. The society made the gift as a large employer, many of whose members become patients.

### National Health Society.

At the recent examinations for Health Visitors, Infant Welfare Workers, etc., held at 53 Berners Street, the headquarters of the National Health Society, the following trained nurses passed successfully:—Miss Margaret D. Marwick, Miss Flora Ethel Mercer and Miss Gladys Sarah Thomas. Miss Susan Wilhelmina Foucar also passed the Sanitary Inspectors' Examination Board.

### Freedom of Bournemouth for Mr. W. C. Clark.

The Mayor of Bournemouth has publicly presented Mr. Walter Child Clark with a casket containing the Council's resolution to confer on him the Honorary Freedom of the Borough in recognition of his munificent services to the hospitals. These include the building of a children's ward and a nurses' hostel and the work Mr. Clark undertook for wounded soldiers during the war.

### Retirement of Sir David Wallace.

Sir David Wallace, K.B.E., C.M.G., President of the Royal College of Surgeons, Edinburgh, is retiring from the active charge of the wards at the Edinburgh Royal Infirmary, to which he was appointed assistant surgeon in 1893. He received the C.M.G. for his services in South Africa in 1900, and was created a K.B.E. in 1920 for his work during the Great War as Commissioner of the Scotch Branch of the British Red Cross.

### Cancer Increasing in France.

Some disquieting statistics indicating the progress in one great district of Southern France have been communicated to the Academy of Medicine by Dr. Rémond, of Toulouse, who has devoted his life to the study of this disease. Particulars rendered to Dr. Rémond by 270 medical men in 649 localities which may be roughly described as being within the Toulouse area show the following progressive increase of cancer in the last four years: 300 cases in 1919; 362 cases in 1920; 585 cases in 1921; 846 cases in 1922—that is, an increase of 546 cases in four years.

### New Hospital at Retford.

The Duchess of Portland performed the opening ceremony of the new Retford and District Hospital. The hospital is situated on a site given by Mr. Denman and comprises three spacious wards—one for men, another for women and a third for children. Each ward is arranged with an open-air verandah facing south, and is so designed as to be capable of indefinite expansion should the needs of the district require. The original cost of the institution was £10,000, but this figure has been largely exceeded by the provision of more complete and up-to-date fittings, electric light and power installation, and the provision of a laundry.

### Public Health in Ireland.

The Irish Nurses' and Midwives' Union has addressed a letter to each candidate for the Dail calling attention to the great importance of securing efficient and adequate health services for the Irish people in the future. The letter asks whether the candidate is prepared to advocate the immediate establishment of a Ministry of Health, and support claims for decent conditions of work and a living wage for all nurses in the public service, all nursing appointments to be whole time and pensionable.

### Retirement of Plymouth Hospital Secretary.

Mr. P. J. Langdon, the secretary of the South Devon and East Cornwall Hospital, Plymouth, is retiring from his post in October. The pension that he has been granted is the recognition of his twenty years' work. A Devonian by birth, Mr. Langdon entered hospital life as assistant secretary of the Hampstead General Hospital. From there he went as secretary to St. Columba's (then the Friedenheim) Hospital in London, where he remained till he was given his present post at Plymouth in 1902. Mrs. Langdon, who is a sister of Mr. Charles Shannon, A.R.A., was formerly on the nursing staff of the hospital.

### International Union against Tuberculosis.

At the annual meeting in Paris of the Council of Directors of the International Union against Tuberculosis it was decided that the following questions should be placed on the agenda of the conference to be held at Lausanne in September, 1924:—Relations between pregnancy and tuberculosis, to be reported on by Prof. Forssner (Stockholm); Do there exist naturally or can there be produced artificially saprophytic forms of Koch's bacillus which might become virulent tuberculosis bacilli? Report by Prof. Calmette (Paris); Effects of the organisation of the anti-tuberculosis campaign in different countries on the decrease in tuberculosis mortality. Report by Sir Robert Philip (Edinburgh).

### Church Contributions to Hospitals.

At the annual council meeting of the Metropolitan Hospital Sunday Fund it was stated that the total of the fund amounted to £84,000 as against £95,000 of the previous year. They had hoped (said Mr. Holland-Martin) to have kept up to a standard of £100,000 or over, but they had not attained this sum, despite the untiring work of the clergy and the splendid assistance given in the publicity of their appeal by the Press. The fact was, he continued, they were heavily down, not only in donations, but in Church contributions. The cause of this deficit was, apparently, the fact that two collections for hospitals were made within one week. That was very trying to the public, and he hoped in another year they would come to some arrangement whereby they could approach the public with a wider gap in the dates of collections.

### Lunacy and Child Mortality in Scotland.

On January 1 of the present year, exclusive of insane persons maintained at home by their natural guardians, there were in Scotland 18,248 insane persons of whom the Board of Control had official cognisance. Of these 2,878 were maintained from private sources, 15,300 by parochial rates, and 70 at the expense of the State. The total on the corresponding date in 1922 was 18,027, thus indicating an increase this year of 221. The Fourth Annual Report of the Scottish Board of Health states that in the three years 1919-21 the rate of infant mortality averaged 94 per 1,000 births in Scotland, as compared with 84 in England and Wales. In both countries a high rate of infant mortality is associated with a high death-rate in children over one and under five years of age. Thus in the experience of Scotland as a whole, in 1915 an infant death-rate of 126.5 was associated with a death-rate at ages one to five of 22.5, while in 1921 an infant death-rate of 90.3 was associated with a death-rate at ages one to five of 12.4.

